# Combination of Colistin and Azidothymidine Demonstrates Synergistic Activity against Colistin-Resistant, Carbapenem-Resistant *Klebsiella pneumoniae*

**DOI:** 10.3390/microorganisms8121964

**Published:** 2020-12-11

**Authors:** Ya-Ting Chang, Tsung-Ying Yang, Po-Liang Lu, Shang-Yi Lin, Liang-Chun Wang, Sheng-Fan Wang, Ya-Ju Hsieh, Sung-Pin Tseng

**Affiliations:** 1 Division of Infectious Diseases, Department of Internal Medicine, Kaohsiung Medical University Hospital, Kaohsiung 807377, Taiwan; yating_iris@yahoo.com.tw (Y.-T.C.); d830166@gmail.com (P.-L.L.); amoe616@gmail.com (S.-Y.L.); 2 Department of Medical Laboratory Science and Biotechnology, College of Health Sciences, Kaohsiung Medical University, Kaohsiung 807378, Taiwan; zegma040899@gmail.com (T.-Y.Y.); wasf1234@kmu.edu.tw (S.-F.W.); 3 Center for Liquid Biopsy and Cohort Research, Kaohsiung Medical University, Kaohsiung 807378, Taiwan; 4 School of Post-Baccalaureate Medicine, College of Medicine, Kaohsiung Medical University, Kaohsiung 807378, Taiwan; 5 Department of Marine Biotechnology and Resources, National Sun Yat-sen University, Kaohsiung 80424, Taiwan; marknjoy@g-mail.nsysu.edu.tw; 6 Center for Tropical Medicine and Infectious Disease Research, Kaohsiung Medical University, Kaohsiung 807378, Taiwan; 7 Department of Medical Imaging and Radiological Sciences, Kaohsiung Medical University, Kaohsiung 807378, Taiwan

**Keywords:** Klebsiella pneumoniae, azidothymidine, caenorhabditis elegans

## Abstract

Carbapenem-resistant Enterobacteriaceae (CRE) is listed as an urgent threat by the World Health Organization because of the limited therapeutic options, rapid evolution of resistance mechanisms, and worldwide dissemination. Colistin is a common backbone agent among the “last-resort” antibiotics for CRE; however, its emerging resistance among CRE has taken the present dilemma to the next level. Azidothymidine (AZT), a thymidine analog used to treat human immunodeficiency virus/acquired immunodeficiency syndrome, has been known to possess antibacterial effects against Enterobacteriaceae. In this study, we investigated the combined effects of AZT and colistin in 40 clinical isolates of colistin-resistant, carbapenem-resistant *K. pneumoniae* (CCRKP). Eleven of the 40 isolates harbored *Klebsiella pneumoniae* carbapenemase. The in vitro checkerboard method and in vivo nematode killing assay both revealed synergistic activity between the two agents, with fractional inhibitory concentration indexes of ≤0.5 in every strain. Additionally, a significantly lower hazard ratio was observed for the nematodes treated with combination therapy (0.288; *p* < 0.0001) compared with either AZT or colistin treatment. Toxicity testing indicated potentially low toxicity of the combination therapy. Thus, the AZT–colistin combination could be a potentially favorable therapeutic option for treating CCRKP.

## 1. Introduction

Antimicrobial resistance (AMR) has been one of the most challenging public health problems during this era of expeditious medical advances. Multidrug-resistant organisms (MDROs) are recognized as an imminent global threat that impose substantial medical burdens and economic costs [1]. Various carbapenem-resistant Gram-negative bacteria (GNB) are listed as being critical and urgent MDRO threats by the World Health Organization and the US Centers for Disease Control and Prevention (CDC) [2,3] because of the rapid evolution of their resistance mechanisms and worldwide dissemination. Globally, the prevalence rates of carbapenem-resistant Enterobacteriaceae (CRE) have been increasing, especially those of *Klebsiella pneumoniae* and *Escherichia coli* [4]. Because of the limited antimicrobial options and an increased risk for horizontal transmission due to the presence of resistance genes in mobile genetic elements, the treatment and containment of CRE infections have become serious dilemmas in daily practice and in terms of infection control [5].

The phenotypic resistance to carbapenems in CRE typically originates from two main mechanisms: (1) the combined effects of β-lactamase(s) and structural mutations, and (2) the production of carbapenemases [6]. Therefore, CRE is frequently categorized into carbapenemase-producing (CP) CRE (CP-CRE) and non-CP CRE (non-CP-CRE). CP-CRE exhibits higher carbapenem minimum inhibitory concentrations (MICs), results in higher mortality, and has an increased risk for resistance transmission [7,8]. CP-*K. pneumoniae* (CPKP) is the most widely reported CRE species, with a much higher and increasing prevalence compared with *E. coli* [9]. Therefore, CPKP is the most extensively studied CRE regarding treatment response to various new and old antibiotics. 

Among the list of “old antibiotics” for emerging MDROs, polymyxin B and E (colistin) are frequently used as the backbone of combination therapy for CRE [10]. In countries or regions where new β-lactam/β-lactamase inhibitors (BLBLIs) are unavailable, colistin resistance in CRE has a significant impact on patient survival [11]. In such a difficult situation, combining colistin with a synergistic agent would be a potential option and feasible approach. Azidothymidine (AZT; 3′-azido-3′-deoxythymidine), and also known as zidovudine, is a thymidine analog used as an antiretroviral agent to treat human immunodeficiency virus/acquired immunodeficiency syndrome (HIV/AIDS). It has been found to possess antibacterial effects against GNB [12,13,14]. Several studies that were conducted to repurpose screens among FDA-approved drugs showed that AZT is a potential candidate for combination therapy of multidrug-resistant GNB, including CRE [14,15,16]. 

In this study, we performed synergistic analyses between AZT and colistin on 40 isolates of colistin-resistant, carbapenem-resistant *K. pneumoniae* (CCRKP) by using the checkerboard method. Meanwhile, an animal model using *Caenorhabditis elegans* was applied to evaluate the in vivo efficacy and safety of combination therapy. To the best of our knowledge, this study is the first to examine AZT–colistin synergism for concomitant colistin- and carbapenem-resistant *K. pneumoniae* isolates. 

## 2. Materials and Methods

### 2.1. Bacterial Isolates

As a part of Taiwan’s nationwide surveillance, 40 isolates of CCRKP were collected between 2013 and 2015 from 11 hospitals in Taiwan [9,17]. Carbapenem resistance was defined as resistance to either imipenem or meropenem, according to the Clinical and Laboratory Standards Institute (CLSI) guidelines [18]. Colistin resistance was defined as MIC > 2 μg/mL, according to the European Committee on Antimicrobial Susceptibility Testing (EUCAST) criteria [19]. Among the 40 isolates, urine was the main source (11/40, 27.5%), followed by sputum (10/40, 25%), stool (4/40, 10%), pus/wound (3/40, 7.5%), abscess (3/40, 7.5%), blood (2/40, 5%), endotracheal aspirate (2/40, 5%), ascites (2/40, 5%), bile (2/40, 5%), and drainage (1/40, 2.5%) (Table 1).

### 2.2. Antimicrobial Susceptibility

The broth microdilution method (Sensititre, Trek Diagnostic Systems, Cleveland, OH, USA) was used to determine the isolates’ susceptibility profiles against 18 antimicrobial agents, according to the CLSI guidelines [18], including β-lactams (ampicillin, ceftazidime, cefazolin, cefepime, cefoxitin, ceftriaxone, imipenem, meropenem, doripenem, ertapenem, cefotaxime, and piperacillin-tazobactam), monobactams (aztreonam), aminoglycosides (amikacin and gentamicin), quinolones (ciprofloxacin and levofloxacin), and folate inhibitors (trimethoprim/sulfamethoxazole). For tigecycline and colistin, the Enterobacteriaceae breakpoints were adopted, according to the Food and Drug Administration (FDA) and EUCAST guidelines, respectively. Furthermore, a standard broth microdilution method was applied in accordance with CLSI guidelines to evaluate the MICs for colistin and AZT. 

### 2.3. Synergistic Analysis

The synergism between AZT and colistin was investigated through the checkerboard method, as described previously [20]. In brief, the bacterial suspension of each strain was prepared in cation-adjusted Mueller–Hinton broth (CAMHB) and was added to wells at a final concentration of 5 × 10^5^ CFU/mL. Two-fold serial dilutions of AZT and colistin were prepared and added to the wells containing bacteria. The synergistic effects were determined according to the fractional inhibitory concentration (FIC) index, which was calculated as follows:

(MIC of drug A tested in combination)/(MIC of drug A tested alone) + (MIC of drug B tested in combination)/(MIC of drug B tested alone).

Synergy is defined as an FIC index ≤ 0.5. An FIC index between 0.5 and 4.0 is interpreted as there being no interaction, and antagonism is defined as an FIC index of >4. 

### 2.4. In Vivo Study

*C. elegans*, strain N2, was used for both in vivo toxicity and nematode-killing assays. Nematode growth medium (NGM) agar plates with bacterial lawns of *E. coli*, laboratory strain OP50, were used as food sources for the maintenance of nematodes at 20 °C. The procedures were carried out as described in our previous study [21]. NGM agar plates were prepared with the following antibiotic concentrations: colistin, 1 μg/mL; AZT, 0.15 μg/mL; a combination of colistin and AZT, 1 and 0.15 μg/mL, respectively. Clinical isolates of CCRKP strain 1336 or *E. coli* OP50 were spread onto the plates. Growth-synchronized L4-stage nematodes (40 for each group) were transferred onto the plates containing different antimicrobial agents and bacterial lawns. All plates were maintained at 25 °C, and the survival of nematodes was recorded daily. For the toxicity assay, treatment plates that were supplemented with a vehicle served as the controls. For the killing assay, the plates containing only a lawn of *E coli* OP50 served as the negative control.

### 2.5. Polymerase Chain Reaction Detection

Polymerase chain reactions were used to detect the presence of extended-spectrum β-lactamase (ESBL) genes (*bla*_SHV_, *bla*_TEM_, *bla*_OXA_, *bla*_CTX-M-G1_, *bla*_CTX-M-G2_, and *bla*_CTX-M-G9_), plasmid-mediated AmpC genes (*bla*_DHA_ and *bla*_CMY_), carbapenemase genes (*bla*_KPC_, *bla*_NDM_, *bla*_IMP_, *bla*_NMC_, *bla*_SME_, *bla_VIM_*, *bla*_SPM-1_, *bla*_GIM-1_, *bla*_SIM-1_, *bla*_IMI_, *bla*_GES_, and *bla*_OXA-48_), *mcr-1* gene and outer membrane porin genes (*ompK35* and *ompK36*) (Table S1) [21]. Reactions were performed using TaKaRa Taq™ (Cat. R001A, Takara Shuzo Co., Ltd., Tokyo, Japan) and prepared in a total volume of 25 μL, in accordance with the instruction manual. All analyses were performed with corresponding positive controls. 

### 2.6. Statistical Analyses

The MIC distributions of colistin and AZT, in combination or alone, were constructed with GraphPad Prism software (v.7.0) and analyzed using paired Student’s *t*-tests.

## 3. Results

### 3.1. Distribution of Resistance Mechanisms and In Vitro Susceptibilities

**Figure 1:**
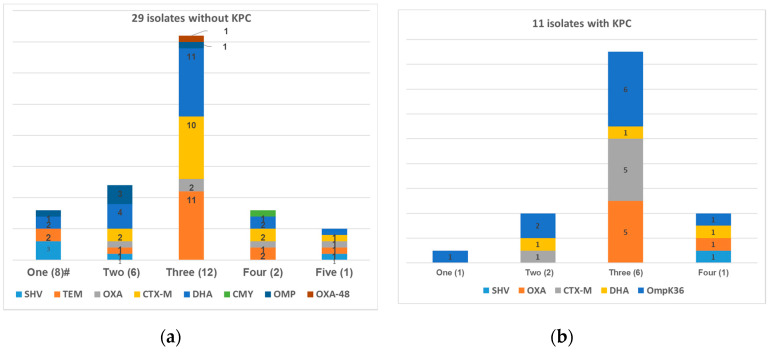
Distribution of the examined resistance mechanisms among the 40 isolates. (**a**) non-KPC producers; #, number of simultaneous resistance mechanisms (number of isolates) (**b**) KPC producers, with one isolate lacking data for outer membrane proteins.

**Figure 2:**
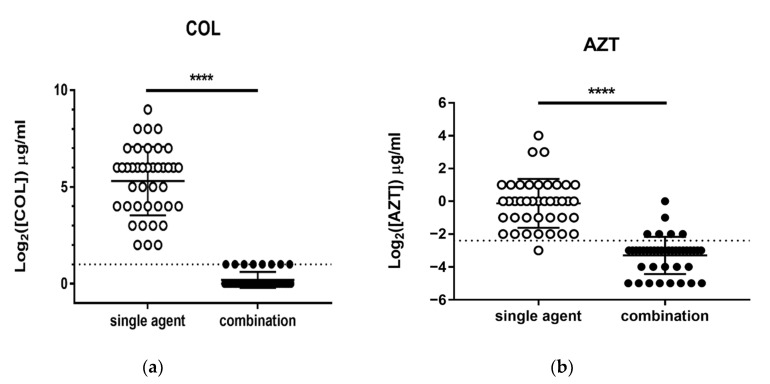
MIC distributions of colistin (COL, hollow circles), azidothymidine (AZT, hollow circles), and their combination (filled circles). (**a**) Dotted line indicates the breakpoint for colistin resistance according to the EUCAST guidelines. (**b**) Dotted line represents the average steady-state concentration of AZT in plasma (0.19 μg/mL). ****, *p* < 0.0001 with the paired *t*-test.

Among the 40 isolates of CCRKP, *Klebsiella pneumoniae* carbapenemase (KPC) was identified in 11 of them and was the only carbapenemase. None of the strains harbored the *mcr1* gene. For non-CP-CCRKP, 51.7% (15/29) had more than three types of resistance mechanisms, which most commonly involved DHA AmpC β-lactamase gene (20 isolates) and ESBL genes (TEM, 17 isolates; CTX-M, 15 isolates) (Figure 1a). It was determined that all except one isolate of CP-CCRKP had lost OmpK36 (Figure 1b). The antimicrobial susceptibilities to 19 common antimicrobial agents are listed in Table 2; tigecycline and amikacin demonstrated higher susceptibilities of 87.5% and 70%, respectively, compared with the other antimicrobials tested. The MICs for colistin ranged from 4 to 12 μg/mL, with the MIC_50_ being 64 μg/mL and MIC_90_ being 128 μg/mL. The MICs for AZT ranged from 0.125 to 16 μg/mL, with the MIC_50_ being 1 μg/mL and MIC_90_ being 2 μg/mL (Table 3). Table 3 also shows the changes to MICs after the two drugs were combined. MICs for both AZT and colistin decreased significantly after combining with the respective drug, ranging from 1 to 2 μg/mL for AZT and 0.03125 to 1 μg/mL for colistin. Figure 2 presents a comparison of the MICs of AZT and colistin as single agents and in combination. After combination, the MIC values of colistin fell below the breakpoint for resistance (MIC ≤ 2 μg/mL) in all strains. Moreover, the mean MIC value of AZT attained an average steady-state serum concentration of 0.19 μg/mL after combination with colistin [22]. 

### 3.2. Checkerboard Analysis and In Vivo C. elegans Toxicity and Killing Assay

To determine the combined effects of colistin and AZT, the checkerboard method was utilized and demonstrated synergistic activity (Table 4). The FIC indexes were ≤0.5 for 100% of both KPC producers and nonproducers, clearly showing synergism in the 40 strains of CCRKP, regardless of carbapenemase production (all KPC in this study). 

**Figure 3:**
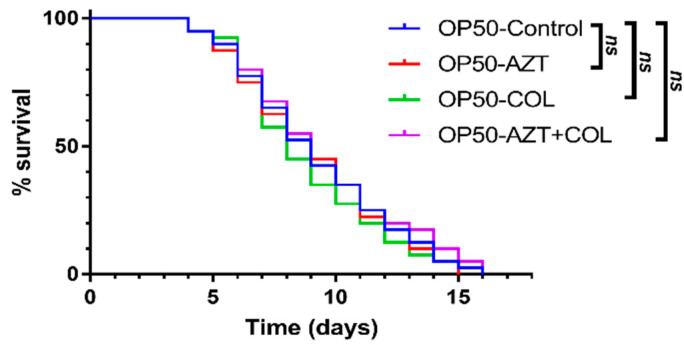
In vivo toxicity testing of colistin, AZT, or colistin/AZT combination using a *C. elegans* model. Nematodes (n = 40, each group) were fed with nontoxic *E. coli* laboratory strain OP50 and supplemented with a vehicle (control), colistin (COL, at 1 μg/mL), azidothymidine (AZT, at 0.15 μg/mL), or a combination (AZT + COL, at 0.15/1 μg/mL). ns, no significance.

**Figure 4:**
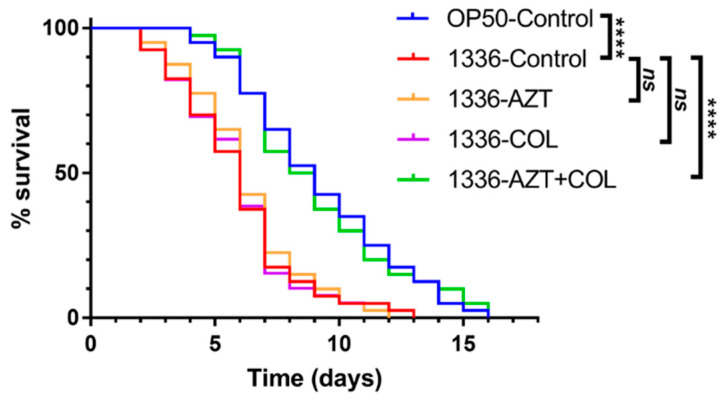
In vivo killing assay of CCRKP strain 1336 using a *C. elegans* model. Forty nematodes were infected with CCRKP clinical strain 1336 (isolated from blood) and supplemented with a vehicle (control), colistin (COL, at 1 μg/mL), azidothymidine (AZT, at 0.15 μg/mL), or a combination (AZT + COL, at 0.15/1 μg/mL). ns, no significance; ****, *p* < 0.0001.

For the toxicity testing of colistin, AZT, and their combination, *C. elegans* were fed with *E. coli* OP50 and treated with different regimens. As shown in Figure 3 and Table 5, no differences were observed between the groups of nematodes treated with colistin, AZT, or a combination thereof compared with the *E. coli* OP50 control group. This result suggests that the combination of AZT and colistin is safe. In addition, the synergistic activity between AZT and colistin was confirmed using the nematode killing assay. *C. elegans* fed with nontoxic *E. coli* OP50 (OP50-control) had a significantly longer median survival time (9 days; *p* < 0.0001) compared with *C. elegans* infected with a clinical strain of *K. pneumoniae* 1336 (1336-control) (Figure 4 and Table 5). There were no significant differences found between the 1336-control and the nematodes treated with either colistin at 1 μg/mL or AZT at 0.15 μg/mL, implying that neither of the antimicrobial agents could rescue the nematodes infected with a CCRKP. By contrast, the infected nematodes that were treated with a combination of colistin and AZT had a medium survival time that was significantly extended, from 6 days to 8.5 days (*p* < 0.0001). A significantly lower hazard ratio (HR) was observed for the nematodes treated with combination therapy (HR, 0.288; 95% confidence interval 0.17 to 0.50; *p* < 0.0001). 

## 4. Discussion

CRE has become an imminent global hazard with increasing prevalence worldwide, leading to increased healthcare costs and higher mortality [23]. Among the carbapenemases with a high risk for transmission, KPC is the most prevalent and widely spread [24]. Despite the introduction of novel BLBLIs on the market in recent years, such as ceftazidime/avibactam, imipenem/relebactam, and meropenem/vaborbactam, polymyxins remain an important agent for the treatment of CRE, especially in regions where the new BLBLIs are unavailable or prevalent with metallo-β-lactamases-producing CRE. Nevertheless, the emerging resistance of CRE against “last-resort” antibiotics, such as colistin, tigecycline, and fosfomycin, has been increasingly reported [11,25,26,27]. The emergence of the colistin resistance gene *mcr1* in CRE is of great concern, especially with its potential to spread into geographical areas beyond China and Southeast Asia, where it is most frequently reported, and into Enterobacteriaceae species other than *E. coli* and *K. pneumoniae* [28,29].

In this study, the *mcr1* gene was not detected in any of the 40 clinical isolates of CCRKP. The extensive resistance primarily comes from the combination of multiple extensive-spectrum β-lactamases or carbapenemase (all KPC), predominantly combined with outer membrane protein deficiency (all Omp36K). AZT alone demonstrated relatively low MICs for the CCRKP (Table 3), and a similar observation was reported by Peyclit et al. for one strain of colistin-resistant, carbapenem-resistant *K. pneumoniae*, with AZT being highly effective at an MIC of 0.104 μg/mL [14]. Among the 12 strains of isolates with relatively higher AZT MICs, ranging from 2 to 16 μg/mL (nine isolates at 2 μg/mL, two isolates at 8 μg/mL, one isolate at 16 μg/mL; data not shown), only two strains harbored KPC, both with MICs at 2 μg/mL. Notably, all KPC-producing strains had lower MICs for AZT (0.25–2 μg/mL). Among the three isolates that exhibited the highest MICs for AZT, two had only one resistance gene (SHV (MIC = 8) and DHA (MIC = 16)), and one had multiple β-lactamase genes (TEM, DHA, and CTX-M (MIC = 8)). This observation is reasonable considering that the resistance mechanisms of AZT appear to involve the loss of thymidine kinase activity and/or poor cell membrane permeation [30,31] instead of the more commonly known mechanisms of β-lactamases. This suggests that AZT could be a favorable therapeutic candidate, even for carbapenemase-producing organisms. 

We demonstrated the evident synergy between AZT and colistin using the checkerboard method (Table 4) and nematode killing assay (Table 5). The synergistic activity of polymyxins and AZT among *E. coli* and *K. pneumoniae* carrying various resistance mechanisms (Table 6) has been reported in several previous studies [32,33,34,35]. The most commonly investigated resistance genotypes are *mcr1* and *bla*_NDM-1_. This study is the first to examine the combined effects of AZT and colistin in Enterobacteriaceae with concomitant colistin and carbapenem resistance. It also bears the important clinical implication that the colistin MIC in these 40 isolates decreased dramatically to a range of 1–2 μg/mL after combination with AZT, which is defined as susceptible by the EUCAST guidelines. AZT is a thymidine analog that acts as an inhibitor of HIV reverse transcriptase. Before being adopted as the first antiretroviral drug for HIV/AIDS, it had been noted to exhibit potent in vitro bactericidal activity against various bacteria of the family Enterobacteriaceae [36]. In Enterobacteriaceae, AZT is phosphorylated by thymidine kinase (when present) to its triphosphate metabolites, which are then incorporated into the bacterial DNA chain as a replication terminator [36]. Nonfermenters, such as *Pseudomonas* and *Acinetobacter* species, are unaffected by AZT because they naturally lack thymidine kinase [37]. In addition to the thymidine kinase levels of the bacteria, AZT susceptibility also correlates to cell permeability. Given that colistin lyses GNB by permeabilizing the outer membrane, it is hypothesized that this mechanism allows AZT to enter the cell at increased intracellular drug concentrations [38]. Lin et al. demonstrated that higher polymyxin B concentrations could increase outer membrane permeability, with simultaneous decreases in AZT MIC [33]. In addition, AZT can synergize with colistin by exerting a shared outer membrane disrupting effect [38]. 

The peak plasma AZT concentration (Cmax) of 1–5 μg/mL is achieved approximately 1 h after ingestion of 200–300 mg of AZT [39,40,41,42]. The pharmacokinetics (PK) of AZT has been most extensively investigated among HIV-infected individuals. In the works of Drew et al. [41] and Burger et al. [40], among HIV-infected patients, a Cmax of 0.73–1.3 and 0.8–1.1 μg/mL could be attained at 0.76 and 0.77 h, respectively. Wattanagoon et al. reported a *C*max of 17.98 μM (4.8 μg/mL) in healthy volunteers in Thailand after a single ingested dose of 300 mg AZT [39]. In agreement with a recent study [34], we found relatively low AZT MICs (0.03125–1 μg/mL) for colistin-resistant *K. pneumoniae* after combination with colistin, indicating that AZT could be a practical therapeutic candidate because of its clinically achievable concentrations. Loose et al. [37] investigated the serum bactericidal activity of combined intravenous (IV) colistin methanesulfonate (CMS) and AZT in colistin-resistant GNB (five isolates of *mcr1*-harboring *E. coli*). The trial was part of a phase 1 randomized, double-blinded study in healthy volunteers receiving multiple doses of IV CMS coadministrated with AZT. Seven volunteers received three IV infusions consisting of the following: first infusion, 4 million international units (MIU) of CMS and 200 mg of AZT; second and third infusions, 2 MIU of CMS and 100 mg of AZT. The study found the combination to be well tolerated, with only transient and manageable gastrointestinal side effects. It also revealed bactericidal, or at least bacteriostatic, activities for all strains tested. The results obtained with our in vivo *C. elegans* toxicity testing model are consistent with those reported by Loose et al. [37]. Although AZT appears to be important in the combined treatment of MDROs, the rapid emergence of stable high-level AZT resistance in Enterobacteriaceae has been well documented, which appears to be related to the loss of thymidine kinase activity [30,43]. Lin et al. demonstrated that the combination of polymyxin B and AZT has superior antimicrobial efficacy and minimizes the emergence of resistance to polymyxins [33]. The two-drug combination significantly increased bacterial killing and remained synergistic for up to 48 h in their study. It also significantly delayed bacterial regrowth compared with either monotherapy.

## 5. Conclusions

This study demonstrates the evident synergy between colistin and AZT in 40 clinical CCRKP isolates, regardless of the presence of carbapenemase. In vivo toxicity testing indicated low toxicity of the two-drug combination. The combined treatment also significantly increased the lifespan of *C. elegans* in the nematode killing assay. According to our findings, AZT could serve as a promising component in a combination regimen for CRE treatment. AZT has many beneficial characteristics, such as intravenous formulation, attainable clinical plasma concentration with effective central nervous system penetration, and known safety profiles for a wide range of populations, such as neonates or children and pregnant women. The AZT/colistin combination could be a potential therapeutic option for the treatment of CCRKP. Future investigation into the optimal dosage and frequency is necessary to achieve clinical efficacy and prevent the rapid emergence of resistance. 

## Figures and Tables

**Table 1:** Sources of the 40 isolates.

Source	No. of Isolates
Urine	11
Sputum	10
Stool	4
Pus/wound	3
Abscess	3
Blood	2
Endotracheal	2
Ascites	2
Bile	2
Drainage	1

**Table 2:** Antimicrobial susceptibilities against 19 common antimicrobial agents.

Antimicrobial Agents	Antibiotic Susceptibility		
	S	I	R
Ampicillin	0.0%	0.0%	100.0%
Ceftazidime	0.0%	2.5%	97.5%
Cefazolin	0.0%	0.0%	100.0%
Cefepime	5.0%	7.5%	87.5%
Cefoxitin	2.5%	2.5%	95.0%
Ceftriaxone	0.0%	0.0%	100.0%
Cefotaxime	2.5%	0.0%	97.5%
Imipenem	2.5%	10.0%	87.5%
Meropenem	7.5%	0.0%	92.5%
Doripenem	7.5%	2.5%	90.0%
Ertapenem	0.0%	2.5%	97.5%
Aztreonam	7.5%	2.5%	90.0%
Piperacillin/Tazobactam	5.0%	0.0%	95.0%
Tigecycline	87.5%	10.0%	2.5%
Ciprofloxacin	2.5%	0.0%	97.5%
Levofloxacin	2.5%	0.0%	97.5%
Gentamicin	32.5%	0.0%	67.5%
Amikacin	70.0%	0.0%	30.0%
Sulfamethoxazole-Trimethoprim	12.5%	0.0%	87.5%

**Table 3:** MIC range, MIC_50_, MIC_75_, and MIC_90_ for colistin, AZT, and colistin/AZT combination.

Agents	MIC (μg/mL)				MIC in Combination (μg/mL)			
	Range	MIC_50_	MIC_75_	MIC_90_	Range	MIC_50_	MIC_75_	MIC_90_
Colistin	4–512	64	64	128	1–2	1	1	2
AZT	0.125–16	1	2	2	0.03125–1	0.125	0.125	0.25

MIC, minimum inhibitory concentration; AZT, azidothymidine.

**Table 4:** In vitro synergistic analysis using the checkerboard method.

Group		Activity of the Combination	FICI Criteria	Total No. (%) of Isolates
CCRKP (n = 40)	KPC-producer (n = 11)	synergy	≤0.5	11 (100.0%)
		No interaction	>0.5–4	0
		Antagonism	>4	0
	non-KPC-producer (n = 29)	synergy	≤0.5	29 (100.0%)
		No interaction	>0.5–1	0
		Antagonism	>4	0

FICI, fractional inhibitory concentration index; KPC, *Klebsiella pneumoniae* carbapenemase.

**Table 5:** Statistical analyses of in vivo *C. elegans* toxicity testing and killing assays.

Test	Group	Median Survival (Days)	*p* Value	Risk Ratio	95% CI	
					Lower	Upper
In vivo toxicity assay	OP50-Control	9	-	1	-	-
	OP50-AZT	9	0.7901	0.934	0.57	1.54
	OP50-COL	8	0.4573	0.827	0.50	1.36
	OP50-AZT + COL	9	0.6177	1.13	0.69	1.87
In vivo killing assay	OP50-Control	9	<0.0001	0.27	0.16	0.47
	1336-Control	6	-	1	-	-
	1336-AZT	6	0.7254	1.097	0.65	1.84
	1336-COL	6	0.8561	0.953	0.56	1.61
	1336-AZT + COL	8.5	<0.0001	0.288	0.17	0.50

OP50, laboratory strain of nontoxic *Escherichia coli* OP50 as a food source for nematodes (representing the negative control); COL, colistin (concentration of 1 μg/mL); AZT, azidothymidine (concentration of 0.15 μg/mL); AZT + COL, combination (concentration of 0.15/1 μg/mL); 1336, clinical isolate of CCRKP; CI, confidence interval.

**Table 6:** Combination therapy against Enterobacteriaceae in different countries.

Species	Country	No.	Resistance Phenotype	Resistance Mechanism(s)	MIC of AZT (mg/L)		MIC of Colistin (mg/L)		Ref.
					Alone	Combination	Alone	Combination	
*K. pneumoniae*	Greece	100	Colistin	NA	0.125–4	0.0625–1	4–128	0.25–16	[34]
*E. coli*	China	9	Colistin and tigecycline	*tet*(X) and *mcr-1*	0.5–4	0.3–1.5	4–8	<0.13–4	[32]
*K. pneumoniae*, *E. coli*, *E. cloacae*	UK	7	Carbapenem	*bla*_NDM-1_	2–4	0.25–16	0.125–1	Reduced 32–256 fold	[35]
*E. coli*	UK	13	Colistin	*mcr-1*	8–64		2–8	Reduced 4–256 fold	[35]
*K. pneumoniae*	Taiwan	40	Colistin and carbapenem	multiple, *bla*_KPC_	0.125–16	0.03125–1	4–512	1–2	Present study

NA: not available; *tet*(X), a unique mobile tigecycline resistance gene [32].

## Supplementary Materials

The following are available online at https://www.mdpi.com/2076-2607/8/12/1964/s1, Table S1: Primers used in for PCR detection of antibiotic resistant genes.
