# Supplementary material for: Combination of Colistin and Azidothymidine Demonstrates Synergistic Activity against Colistin-Resistant, Carbapenem-Resistant Klebsiella pneumoniae
Source: Microorganisms. 2020 Dec 11;8(12):1964. doi: 10.3390/microorganisms8121964 (PMC7764370; doi:10.3390/microorganisms8121964)
Supplement: Supplementary file 1 [file microorganisms-08-01964-s001.pdf]

Table S1. Primers used in for PCR detection of antibiotic resistant genes.

Target gene	Primer sequence (5′→3′)		Tm (℃)	Amplicon size (bp)
	Forward	Reverse		
**ESBLs genes**				
*bla*SHV	GGGTTATTCTTATTTGTCGC	TTAGCGTTGCCAGTGCTC	52	927
*bla*TEM	GACAGTTACCAATGCTTAATCA	ATAAAATTCTTGAAGACGAAA	55	1080
*bla*CTX-M-G1	GGTTAAAAAATCACTGCGTC	TTGGTGACGATTTTAGCCGC	55	864
*bla*CTX-M-G2	ATGATGACTCAGAGCATTCG	TGGGTTACGATTTTCGCCGC	55	866
*bla*CTX-M-G9	ATGGTGACAAAGAGAGTGCA	CCCTTCGGCGATGATTCTC	55	870
*bla*OXA-1	ACACAATACATATCAACTTCGC	AGTGTGTTTAGAATGGTGATC	45.6	812
*bla*OXA-2	TTCAAGCCAAAGGCACGATAG	TCCGAGTTGACTGCCGGGTTG	52	701
*bla*OXA-9	CGTCGCTCACCATATCTCCC	CCTCTCGTGCTTTAGACCCG	52	313
*bla*OXA-10	CGTGCTTTGTAAAAGTAGCAG	CATGATTTTGGTGGGAATGG	46	652
**Plasmid-mediated AmpC genes**				
*bla*DHA	CTGATGAAAAAATCGTTATC	ATTCCAGTGCACTCAAAATA	55	1136
*bla*CMY	CTGCTGCTGACAGCCTCTTT	TTTTCAAGAATGCGCCAGGC	55	1109
**Carbapenemases genes**				
*bla*KPC	ATGTCACTGTATCGCCGTCT	TTTTCAGAGCCTTACTGCCC	52	893
*bla*NDM	GGTTTGGCGATCTGGTTTTC	CGGAATGGCTCATCACGATC	54	1133
*bla*VIM-1	TTATGGAGCAGCAACCGATGT	CAAAAGTCCCGCTCCAACGA	52	920
*bla*VIM-2	AAAGTTATGCCGCACTCACC	TGCAACTTCATGTTATGCCG	50	865
*bla*IMP-1	TGAGCAAGTTATCTGTATTC	TTAGTTGCTTGGTTTTGATG	50	738
*bla*IMP-2	GGCAGTCGCCCTAAAACAAA	TAGTTACTTGGCTGTGATGG	50	737
*bla*NMC	GCATTGATATACCTTTAGCAGAGA	CGGTGATAAAATCACACTGAGCATA	52	2158
*bla*SME	AGATAGTAAATTTTATAG	CTCTAACGCTAATAG	42	1138
*bla*SPM-1	CCTACAATCTAACGGCGACC	TCGCCGTGTCCAGGTATAAC	54	650
*bla*GIM-1	AGAACCTTGACCGAACGCAG	ACTCATGACTCCTCACGAGG	54	748

*bla*SIM-1	TACAAGGGATTCGGCATCG	TAATGGCCTGTTCCCATGTG	51	569
*bla*IMI	ATAGCCATCCTTGTTTAGCTC	TCTGCGATTACTTTATCCTC	50	818
*bla*GES	GTTTTGCAATGTGCTCAACG	TGCCATAGCAATAGGCGTAG	52	371
*bla*OXA-48	CAAAGGAATGGCAAGAAAACAAAA	GCGCAGCCCTAAACCATCC	55	798
**Colistin resistance gene**				
mcr-1	CGGTCAGTCCGTTTGTTC	CTTGGTCGGTCTGTAGGG	52	309
**Outer membrane porin genes**				
ompK35	ATCACGAAGGGGTGTACTGC	GGATGGAAAGATGCCTTCAG	55	1225
ompK36	GCGAGGTTAAACCGGACATA	TGCAGCACAATGAAATAGCC	55	1272
